# Pro-environmental behavior and smartphone uses of on-campus engineering students in Xi’an, China

**DOI:** 10.1371/journal.pone.0259542

**Published:** 2021-11-04

**Authors:** Tao Qiang, Honghong Gao, Xiaoli Ma

**Affiliations:** 1 School of Materials & Chemical Engineering, Xi’an Technological University, Xi’an, China; 2 Decarbonization Hub, Xi’an Technological University, Xi’an, China; 3 School of Mechatronic Engineering, Xi’an Technological University, Xi’an, China; 4 School of Science, Xi’an Technological University, Xi’an, China; Indian Institute of Technology Patna, INDIA

## Abstract

**Background:**

The usage status, waste electrical and electronic equipment (WEEE) related to the smartphones of on-campus engineering students should be studied. Furthermore, the correlations between their smartphone profiles with energy consumption and environmental knowledge should be understood make measures improve their environmental behaviors.

**Methods:**

Pro-environmental behavior and smartphone uses of the on-campus engineering undergraduates in Xi’an, China, were investigated with a self-designed questionnaire anonymously. The energy-saving activities they participated in and their e-waste treatment patterns were analyzed.

**Results:**

Most of the respondents had a smartphone with large screen and high battery capacity, which also had long standby/usage time and frequent charging. Average daily power consumption of one smartphone was estimated to be 6.475 Wh. The surveyed undergraduate students changed their smartphones frequently, which produced large quantities of WEEE annually.

**Conclusion:**

Most on-campus students treated their waste smartphones in the proper ways. However, some of them were short of environmental knowledge about their smartphones. Some measures were suggested to improve their environmental concerns. The findings will help the on-campus engineering undergraduates in China to use their smartphones rationally and to deal with their waste smartphones appropriately.

## Introduction

The past two decades has witnessed a globally sharp increase in the production and consumption of electrical and electronic equipment (EEE), such as mobile phone, laptop, tablet and desktop computer [1]. Thus, there is great surges in both the EEE using and the resultant waste electrical and electronic equipment (WEEE) all over the world [2]. Take mobile phones in China as an example, mobile Internet users had reached to 1,007 million by June 2021, which accounts for 99.6% of the total Chinese Internet users, according to the latest statistical report from China Internet Network Information Center (CNNIC) [3]. In this case, how to use the personal mobile devices among young generation rationally [4], becomes an important issue as that how to deal with these waste mobile devices appropriately.

Today, smartphones become a particularly appealing platform for all the on-campus college students and other young generation [5]. The importance of smartphones in on-campus students’ life has led observers to speak of a Mobile Youth Culture, which has the features of fashion, popularity and time poverty [6]. Undergraduate students are early adopters of new EEE [7], which has been an essential part of life for on-campus students [8]. Their smartphones are rarely out of reach whether the setting is a college classroom, library, recreation center, cafeterias or dormitory [9]. Smartphones are central to undergraduate students’ lives [10], since they keep them constantly connected with their friends, family and the Internet. These mobile devices have become a learning tool with great potential in both classrooms and outdoor learning [11]. In some cases, smartphones were used as experimental tools to offer inspiring possibilities for science education [12].

However, ubiquitous smartphone usage has brought some far-reaching influences on university students’ emotions [13], consumption attitude and lifestyle [4, 6]. Some issues, such as the end-of-life waste from smartphones and their associated equipment, have aroused great concerns from the whole society [14, 15]. Previous research concluded that waste electrical and electronic equipment (WEEE) is one of the largest growing waste streams globally [16]. Conscious attention has been given to the effects of frequent smartphone usage on college students’ grade point average, anxiety, satisfaction with life [9, 13], their mental and physical health [9], as well as the disposal behavior and environmental awareness [17]. The possibilities of negative impacts of smartphones to the compulsive user has also been addressed [18–20].

All over the world, environmental problems rooted in human behaviors are the major obstacles to sustainable development. To a certain country, how to appropriately promote residents’ pro-environmental behaviors determines whether or not to mitigate environmental problems [21]. Undergraduate students are a special community in the whole society. Politics- and science-oriented educations have different impacts on their pro-environmental behaviors [22]. The profile and usage status of their smartphones, their environmental knowledge and behaviors, should be paid more attention [23]. It is reported that, there are clear differences between factors influencing community’s pro-environmental behavior, such as gender, years on campus, workplace or in domestic [24–27], even regional Characteristics and policies [28]. In this case, some actions should be taken to measure their pro-environmental initiatives and behavior as a first step [24, 29]. Some research has been made to investigate the pro-environmental behaviors in China, such as residents’ express waste recycling behavior [30], road freight transportation [31], smartphone dating applications on sexual risk behaviors [32], and Internet use on pro-environmental behavior [33]. To our knowledge, however, few efforts have been made to date to investigate their environmental knowledge and behaviors with respect to their smartphone usage and the difference in environmental attitudes and behaviors to gender.

In this sense, this work aims to investigate undergraduates’ smartphone profile and usage status in Xi’an, China. Then, their environmental knowledge and behaviors related to their smartphones will be analyzed, in terms of the respondents’ gender. WEEE and energy consumption from their daily smartphone usage will be evaluated theoretically to measure their pro-environmental behaviors. Some reasonable measures will be proposed to update their environmental behaviors.

## Material and methods

### Questionnaire design

On-campus undergraduates’ smartphones usages and their pro-environmental behavior were measured with a questionnaire in this research. Totally, there are 11 questions including in the questionnaire designed by ourselves, which is focused on undergraduates’ smartphone profiles and usage status (Q1-Q6), as well as their environmental knowledge (Q7-Q9) and behaviors (Q10-Q11), respectively. Questions 7–9 were measured with a 5-point Likert scale [34]. Respondents were asked to indicate their agreement level with a declarative statement: Strongly Agree (SA), Agree (A), Unsure (U), Disagree (D) and Strongly Disagree (SD). The detailed 11 questions and their individual choices were tabulated in Table 1.

**Table 1:** Questions and scale for answers in this survey.

No.	Question	Unit of variable	Choices
**Q1**	Screen size of your mobile phone	Inch	≥5 () 4–4.9 () 3–3.9 () <3 ()
**Q2**	Battery capacity	mAh	≥2500 () 2000–2499 () 1500–1999 () 1000–1499 () 800–999 () <800 ()
**Q3**	Frequency of charging	—	At any time () 3 times or more per day () 2 times per day () once per day () 2 days or more per charge ()
**Q4**	On-time	hr./day	<8 () 8–11.9 () 12–15.9 () 16–19.9 () ≥20 ()
**Q5**	Usage time except voice call	hr./day	<1 () 1–2.9 () 3–4.9 () 5–6.9 () ≥7 ()
**Q6**	Average lifespan	Year	<1 () 1–1.9 () 2–2.9 () 3–3.9 () ≥4
**Q7**	Much energy and resources will be consumed during the manufacturing process of digital devices.	—	Strongly agree () Agree () Unsure () Disagree () Strongly disagree ()
**Q8**	Production process of digital devices will cause serious environmental pollution.	—	Strongly agree () Agree () Unsure () Disagree () Strongly disagree ()
**Q9**	High-value resources from the waste digital devices are easily recycled.	—	Strongly agree () Agree () Unsure () Disagree () Strongly disagree ()
**Q10**	What is your preferable treatment pattern for waste mobile phones?	—	Stored at home () Gifts for others () Sold to the peddler and second-hand market () Recycled by Old-for-New activity () Thrown away as ordinary garbage ()
**Q11**	What kind of energy-saving activity have you have most participated in?	—	Development of advanced materials () Design, development of novel products () Promotion of energy-saving ideas () Old-for-new activity () Recycling of waste mobile phone ()

#### Pilot test of and revision to the questionnaire

The undecided questionnaires were distributed to 50 undergraduates with their consent to test its reliability and validity through an anonymous pre-survey in Xi’an Technological University (XATU), China, on Oct. 11, 2019. These figures are not included in the final results of this research. The questions and the given choices of answers were carefully revised, on the basis of the feedback information. The respondents’ gender was also included in the final version of this questionnaire.

### Data collection

The self-reported questionnaire survey was conducted on Nov. 18–22, 2019. Totally, 280 undergraduates from 5 universities consented to participate in this anonymous survey, including Shaanxi Normal University (SNNU), XATU, Xidian University, Xi’an Jiaotong University (XJTU), and Xi’an Medical University (XMU). There were 56 students from each university. Finally, there are 266 complete and valid answer sheets returned. Among them, 50% comes from male students and other 50% comes from female.

### Data analysis

The collected data were analyzed, according to the respondents’ demographics. A model was established to estimate the daily average power consumption of a smartphone, on the basis of the survey results and 2 assumptions.

## Results and discussion

### Smartphone profiles

**Fig 1:**
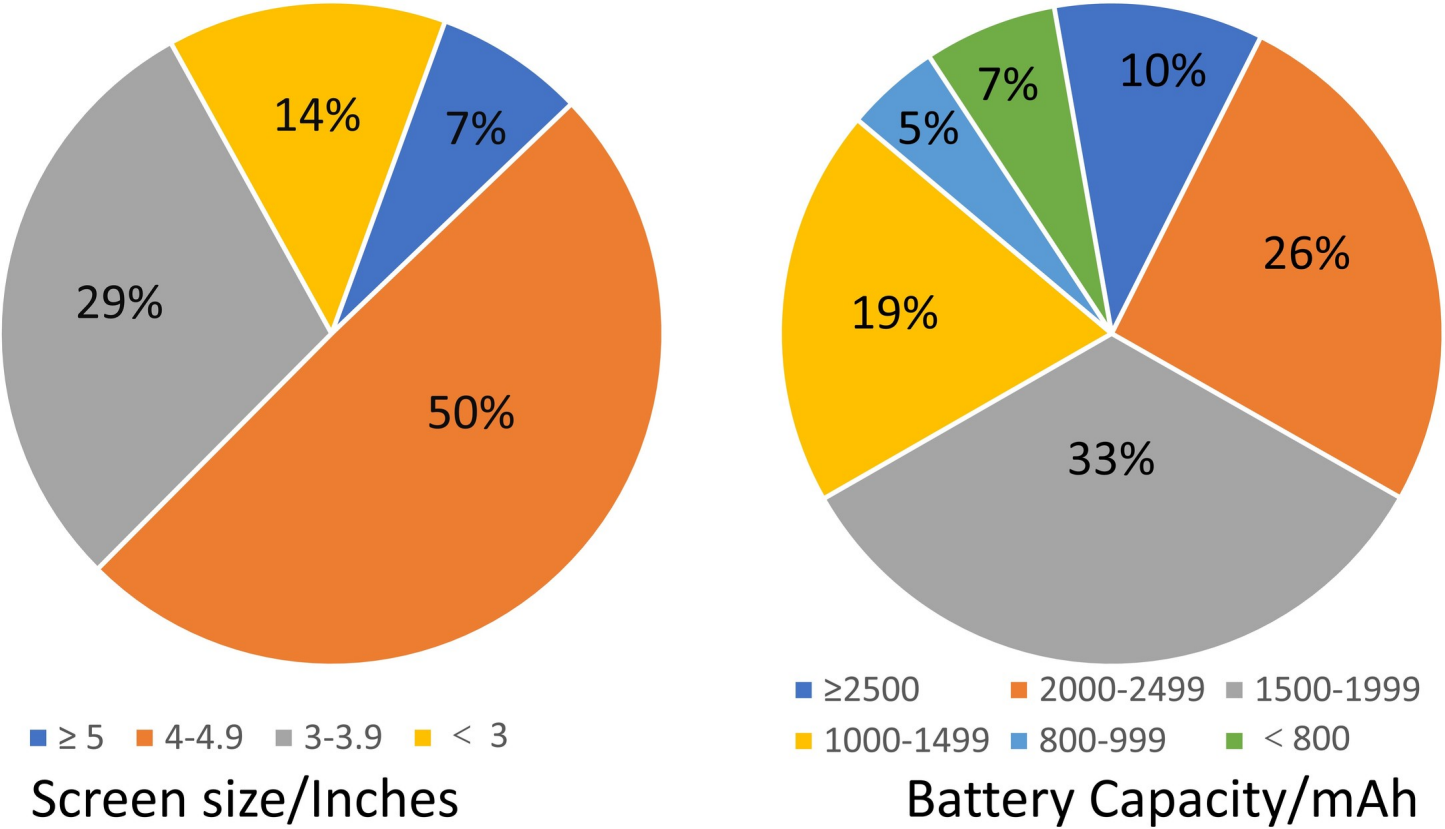
Hardware parameters of the undergraduates’ smartphones.

**Fig 2:**
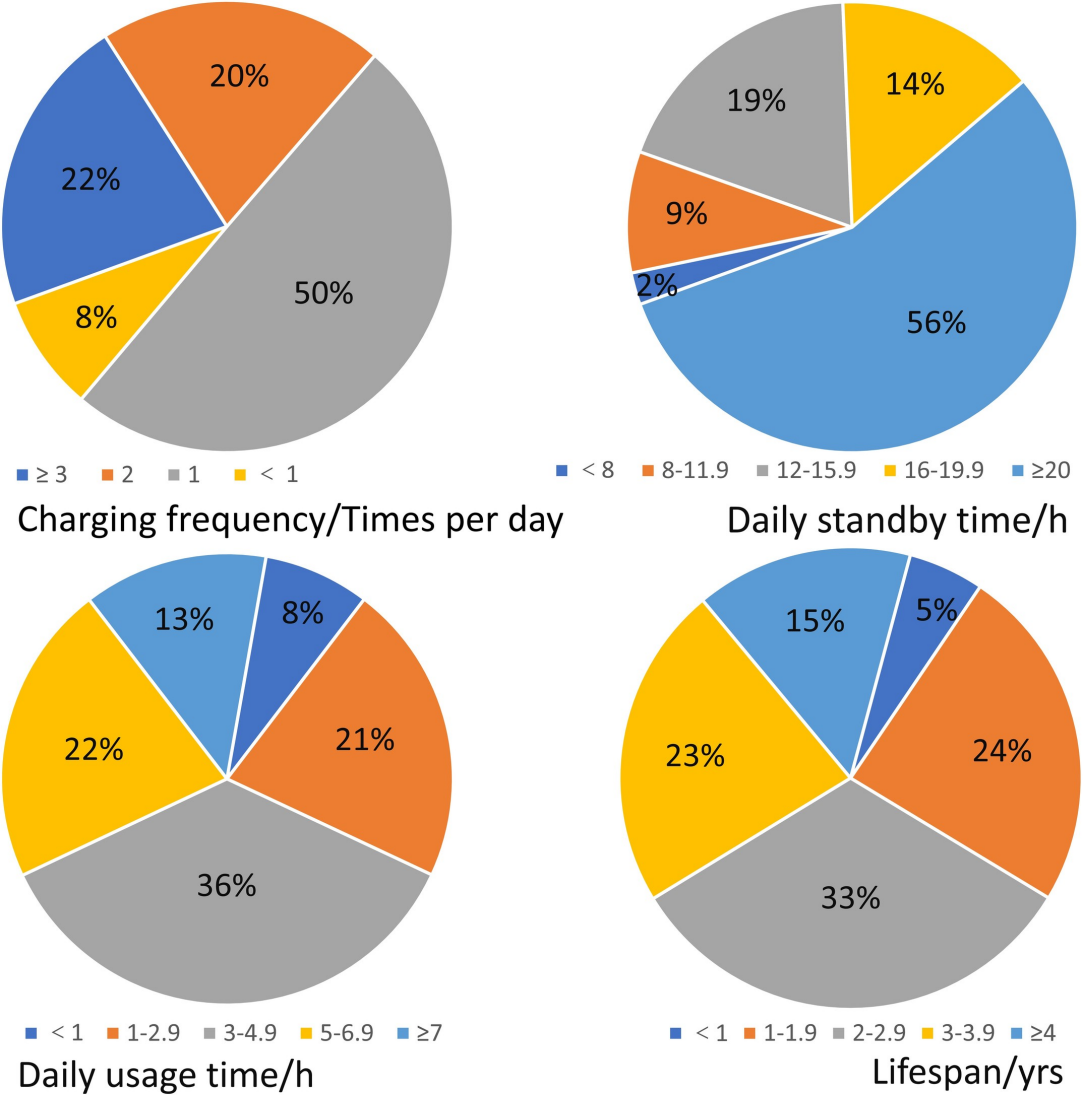
Usage profiles of the undergraduates’ smartphones.

In the era of mobile communication, texting and phoning are not the predominant services any more for a smartphone with various advanced functions [35]. Undergraduates use their fashionable smartphones to socialize with friends, to access to news and information, to take notes in class, to read, to shop online, to amuse themselves after class, even to kill time. Different usage patterns of smartphones reflect different lifestyles of their owners. All the undergraduates who participated in the survey self-reported that they had their own smartphones. The hardware parameters (i.e., screen size and battery capacity) and usage profiles (i.e., charging frequency, daily standby time, daily usage time, and lifespan) of their smartphones, are shown in Figs 1 and 2, respectively.

More than half of the surveyed students have a fashionable smartphone with large screen (≥ 4 inches, 57%), or high battery capacity (≥ 1500 mAh, 69%). On the contrary, smartphones with small screen (< 3 inches) and low battery capacity (< 800 mAh) are only 14% and 7%, respectively. More female students had a smartphone with large screen size than that of the male students.

Nearly 80% of the surveyed students charge their smartphones more than once per day. Among their smartphones, 56% of them had the standby time more than 20 h per day. Only 2% of them had the standby time less than 8 h per day. As far as daily usage time is concerned, 71% of the undergraduates use their smartphones more than 3 h per day. To some sense, they have a certain tendency to use their smartphone irrationally, compulsively and addictively [10, 36]. Using smartphone for a long time every day, particularly day after day, may have the potential negative effects on their memory, sleep, concentration and academic performance [10, 37, 38]. On the whole, one third of the surveyed smartphones had the lifespan of 2 to 3 years, which is close to the previous findings [39, 40]. However, nearly 30% of the surveyed smartphones had a short lifespan (less than 2 years). One reason lies in that, undergraduates frequently upgrade smartphones, due to its relatively short lifespan, fashion obsolescence and their desires for emerging functions [41].

It is found that smartphone with a larger screen also had a higher battery capacity, which was confirmed by the statistical results (Pearson coefficient = 0.605, significant correlation at the 0.01 level, 2-tailed). Such smartphone usually costs much money and consumes more energy than the traditional non-smart counterpart does [14]. There are 50.2% of the smartphones with both large screen (≥ 4 inches) and high battery capacity (≥ 1500 mAh). The respondents (28.3%) charge their smartphones frequently (> 2 times per day). More than 70% of them uses their smartphones more than 3 hours per day. As far as lifespan is concerned, 29.5% of them has a short lifespan (less than 2 years).

**Fig 3:**
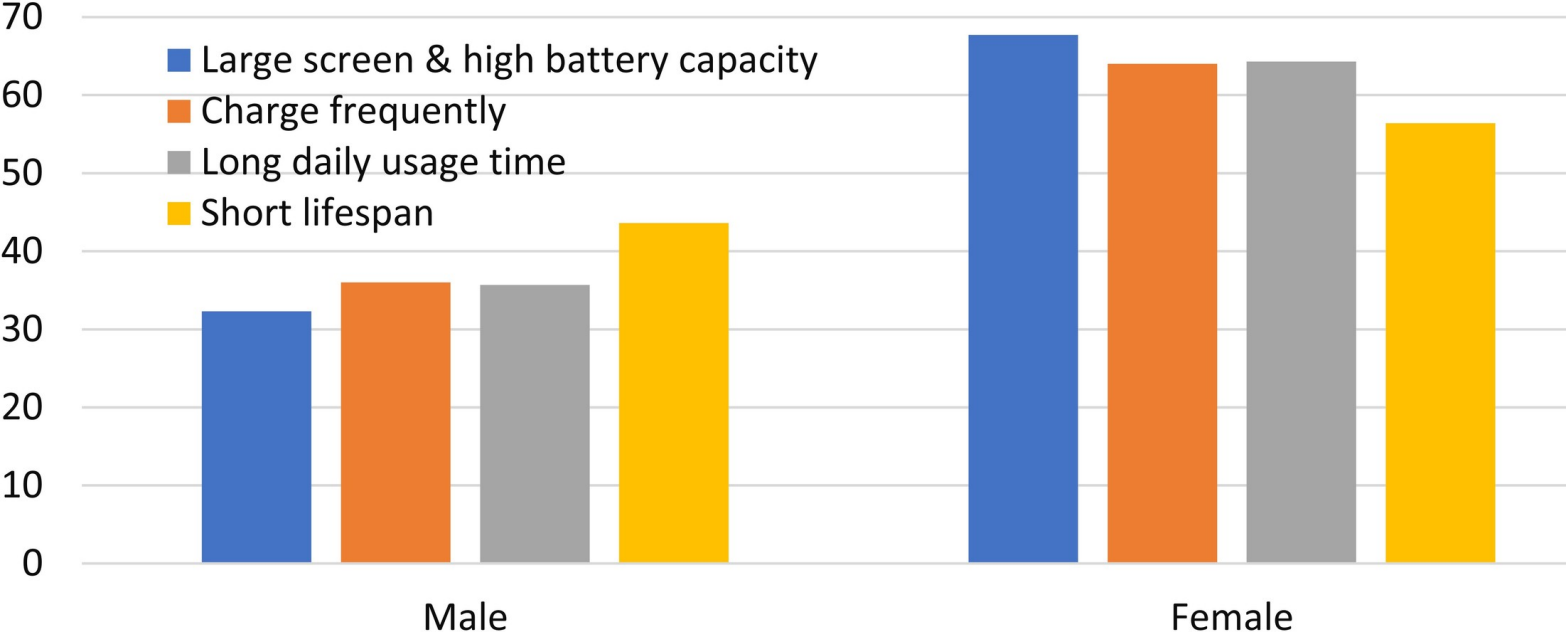
Gender difference of the undergraduates with fashionable smartphones.

What’s more, it is necessary to analyze these parameters from the viewpoint of students’ demographics. Fig 3 shows the gender difference of the students who own fashionable smartphones with both large screen and high battery capacity, charge them frequently, use them for a long time daily, and change them frequently.

Female students are majority of the ones who own a smartphone with both large screen and high battery capacity. Also, the cases of frequent charging, long time usage daily, and frequent changing their fashionable smartphones show the same trends. For these female students, the distinct smartphone usage patterns should be attributed to the advanced hardware facilities and their personal psychological characteristics [42]. An appeal should be made to undergraduates, especially female students, for prolonging the lifespan of their smartphones, in order to save energy, conserve resources, and reduce WEEE [23, 39].

Overall, the surveyed students had a serious reliance on their smartphones during their daily on-campus activities. They should be encouraged to self-monitor their smartphone usage and reflect upon it critically [9], in order to develop healthy habits of green consumption and sustainable lifestyles [43–45].

### Undergraduates’ environmental knowledge

**Fig 4:**
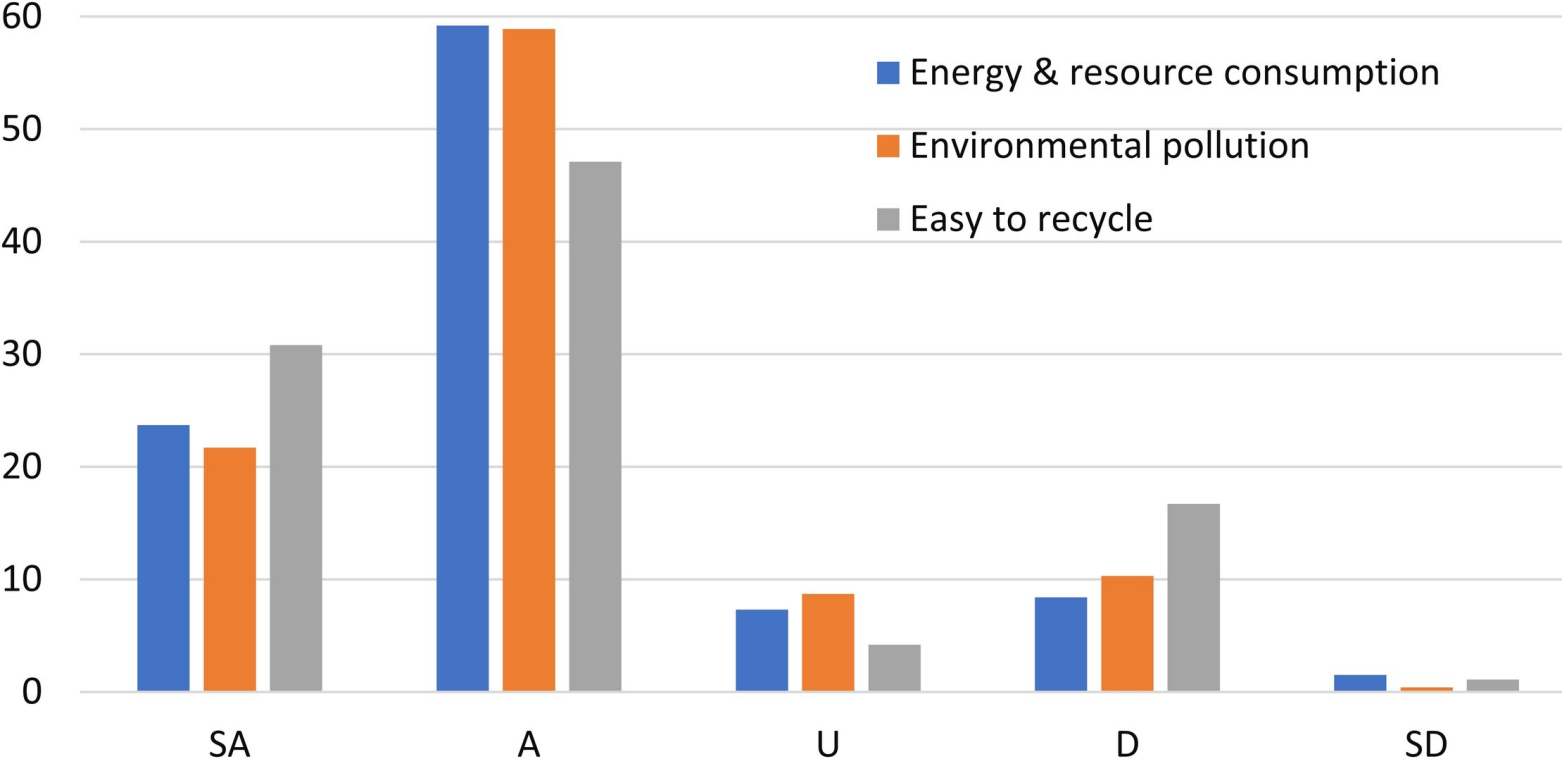
Undergraduates’ environmental knowledge about their smartphones.

Undergraduates’ environmental knowledge about their smartphones was examined with a 5-point Likert scale. Their responses to Questions 7–9 are shown in Fig 4. Most of the respondents (82.9%) agree that smartphone manufacture will consume large amounts of energy and raw materials, which shows that they have the consciousness of energy crisis and raw materials shortage. However, nearly 10% of the surveyed students think that the process does not need large amounts of energy and raw materials.

The reason is that they might take it for granted that, smartphone is just a small portable electronic product, compared with some bulky industrial equipment. So, small amounts of energy and raw materials would be enough during its production phase. In fact, a smartphone is assembled with printed circuit boards (PCB), liquid crystal display, battery, charger, plastic housing unit, and other accessories. Large amounts of raw materials, such as semiconductors, various metals and plastics, is essential for mobile phone production [46]. At the same time, manufacture of a mobile phone will consume large amounts of energy, due to the energy-intensive manufacturing of PCB [47].

There are 10.7% of the respondents believe that the manufacture of a smartphone does not produce a lot of environmental pollutants. Fortunately, most undergraduates in this survey (80.6%) think that, the smartphone production process will cause serious environmental pollution, which indicates that they have strong environmental awareness. A smartphone contains various different substances, including toxic metals such as lead, arsenic, cadmium, hexavalent chromium and flame retardants used in the plastics. So, environmental pollutions and its subsequent environmental impacts from producing smartphones cannot be overlooked [47].

WEEE is one of the fastest-growing pollution problems on college campus, which has the serious potentials to environmental pollution and threats to human health [41]. Materials recycling from WEEE can lead to a 50% reduction of environmental impacts [47, 48]. Nearly 80% of the respondents believe that it is easy to recycle some high-valued materials from WEEE. The other surveyed undergraduates (17.8%) think that it is not easy to do that. The efficient recycling of electronic scrap has been rendered indispensable, which should be regarded as a major challenge for today’s society [49]. WEEE consists of various PBC, metals and plastics, which, for now at least, are not easy to recycle [50, 51]. The informal recycling of e-waste will lead to serious pollution [52]. Advanced technologies and appropriate equipment are essential for recycling of high-valued materials from WEEE [50]. It shows that some of the undergraduates are unfamiliar with WEEE recycling techniques.

**Fig 5:**
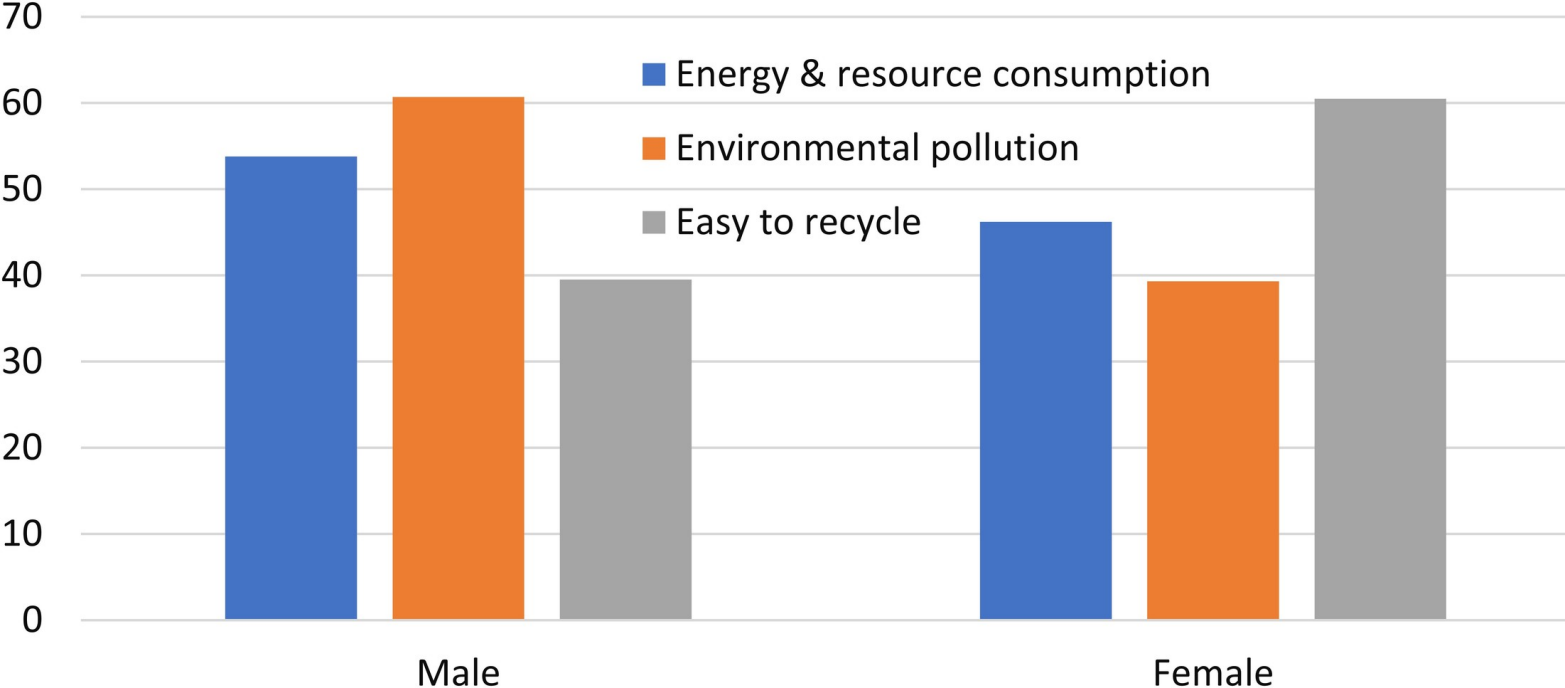
Gender difference of the undergraduates who are short of environmental knowledge about smartphones.

Overall, some surveyed undergraduates are considered lack of the environmental knowledge about smartphones, according to the above results. Further analysis of energy and resource consumption, environmental pollution and recycling, according to their demographics, are shown in Fig 5. Here, more male students had correct ideas about energy and resource consumption, and environmental pollutants than their female peers, which is consistent with the previous findings [53]. In fact, male students also had correct ideas about WEEE recycling, due to inverse problem of Q9.

In practice, WEEE treatment is much more complicated, in contrast to the well-established recycling of metallic scrap, since they contain many different components integrated into each other. The harm to the environment, workers, and local residents is inevitable during the process of WEEE treatment, due to the release of dioxins, furans, and heavy metals [54]. The efficient recycling of waste smartphones is not only a challenge for the recycling industry, but also often a question of as-yet insufficient collection infrastructures and limited collection efficiencies, and a considerable lack of the consumer’s awareness for the potential of recycling electronics for the benefit of environment and the human health, as well as for savings in energy and raw materials [49]. Undergraduates should be instructed to improve their environmental knowledge during their college period [43, 45].

### Undergraduates’ environmental behaviors

#### Treatment patterns for obsolete smartphones

Waste smartphones represent the most valuable EEE in the main waste stream, due to large quantity, high reuse/recovery value and fast replacement frequency [17]. According to the lifespan of the surveyed smartphones, 84.8% of the surveyed students will at least produce a waste phone during their four-year college period, and 29.5% of them will even generate more than 2 retired smartphones. So, undergraduates should be instructed to treat their waste smartphones in a proper way.

**Fig 6:**
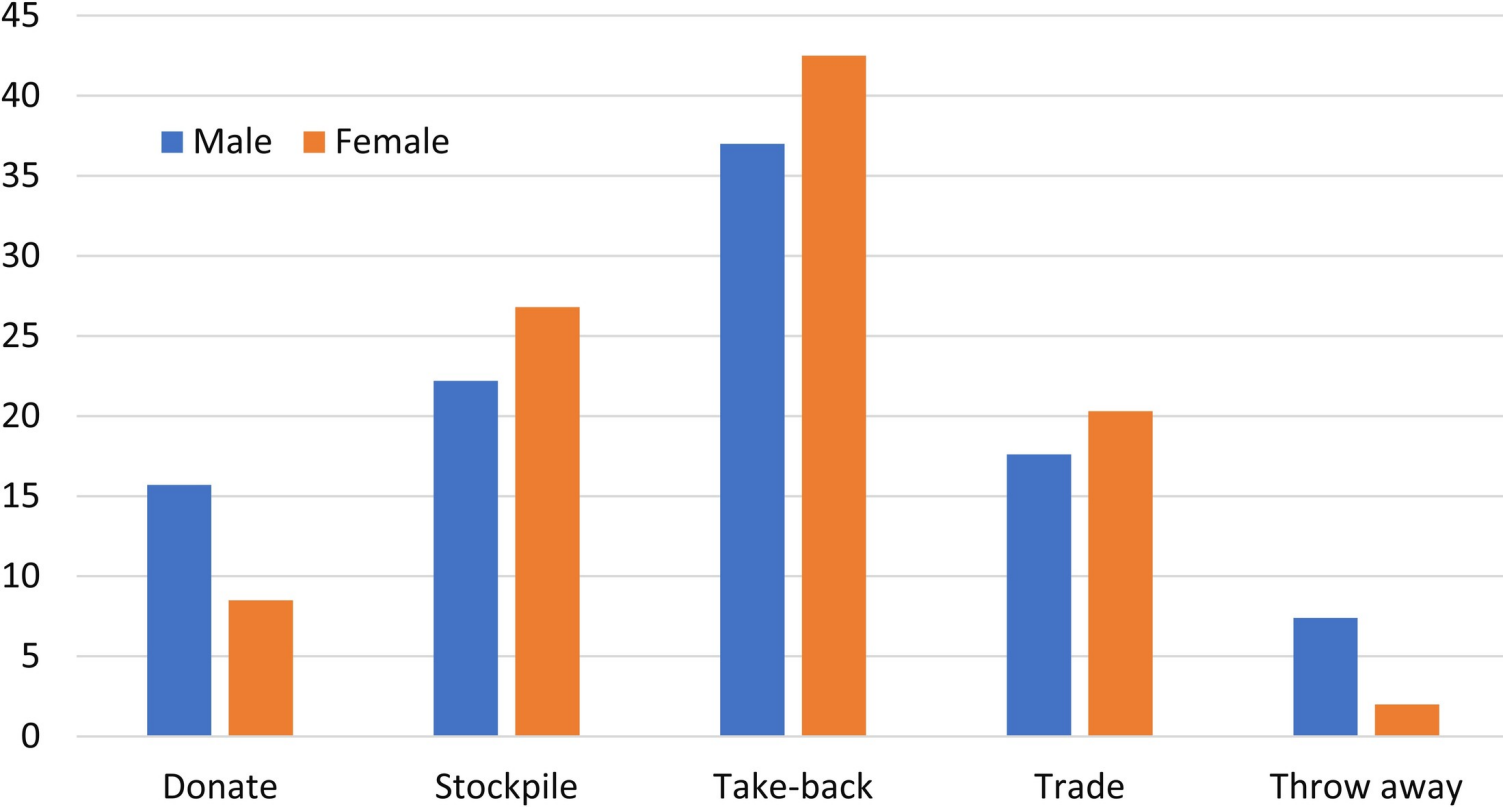
College students’ treatment patterns of the waste smartphones.

Undergraduates’ treatment patterns of the discarded smartphones are shown in Fig 6. Take-back service is the most favorable way by both male and female students, followed by stockpiling at home and trading with the peddler/second-hand market. It is different from Ongondo’s results [55]. They report that stockpiling of unwanted electrical and electronic products is common in both USA and other developed economies. In this survey, most students treated their waste smartphones in the proper ways. Most of their waste smartphones can be recycled and reused. Only 4.2% of the respondents throw their retired smartphones away as general garbage. In this case, the real amount of WEEE from undergraduates’ smartphone was overestimated, according to their smartphones’ lifespan.

There is a distinct difference between male and female students on their treatment patterns of waste smartphones, as presented in Fig 6. Female students are more actively involved in the first three treatment patterns (i.e., take-back service, stockpiling them at home and trade with the peddler/second-hand market) than their male peers.

**Fig 7:**
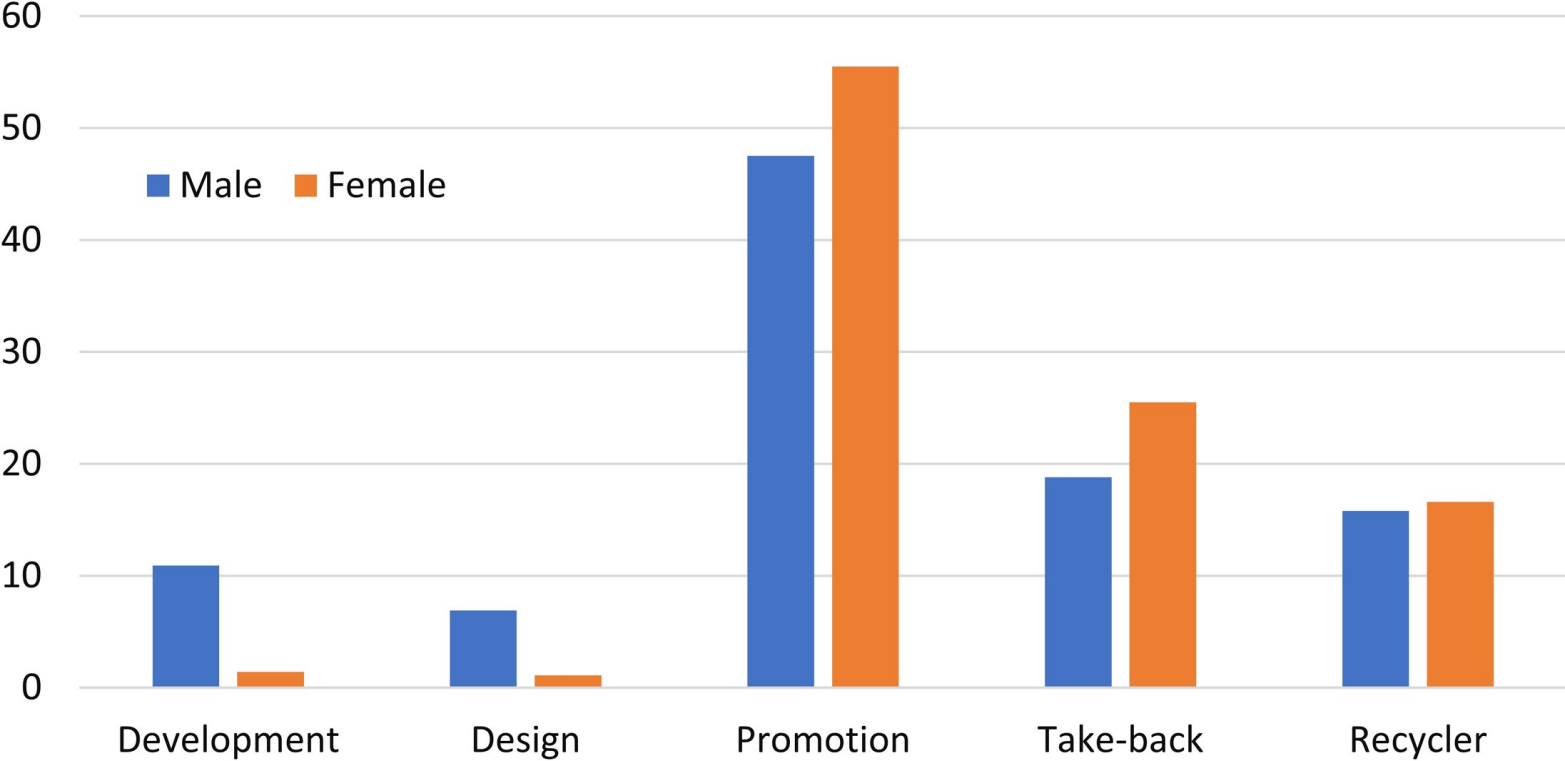
Energy-saving activities for the respondent undergraduates. Here, propaganda means promotion.

*Energy-saving activities*. The energy-saving activities that on-campus undergraduates participated in are shown in Fig 7, according to the answers to Question 11. Promotion of energy-saving ideas (Propaganda in Fig 7) is the most favorite energy-saving activity for both male and female undergraduates. They participated in the take-back service (Take-back in Fig 7) and trade with the peddler/second-hand market (Trade in Fig 7) as their second and third energy-saving choices. Here, female students are more actively involved in the first three energy-saving activities than males. The reason is attributed to that, women express slightly greater environmental concern than men [56].

On the contrary, the on-campus participants of advanced materials development (Development in Fig 7) and of novel products design (Design in Fig 7) are much less than the first three ones. Undergraduates should be encouraged to participate in these on-campus practices, trainings, or internships, as much as possible, especially for female students.

The current WEEE recycling technology is still in its infancy on an industrial scale [50, 51]. Nowadays, only developed countries have conventions, directives, and laws to regulate their disposal, on the basis of extended producer responsibility [54]. On the individual level, WEEE recycling behavior is also strongly influenced by usage behaviors [57], recycling convenience, potential toxicity knowledge of WEEE, prior recycling experience, gender and marital status, education and age, as well as ethnicity [58]. The key to successful WEEE treatment is to develop eco-design devices, collect WEEE properly, recover and recycle material by safe methods, dispose of WEEE by suitable techniques, forbid the transfer of used electronic devices to developing countries, and raise citizen’s awareness of WEEE [41]. Several tools including life cycle assessment (LCA), material flow analysis (MFA), multi criteria analysis (MCA) and extended producer responsibility (EPR) have been developed to manage WEEE, especially in some developed countries [41]. Sustainable WEEE disposal is hopefully industrialized with more suitable methods and advanced equipment in developing countries in the future.

#### Daily power consumption

Smartphone is one of the battery-powered devices that have some notable characteristics with respect to energy consumption [5]. It is important and meaningful to estimate the daily power consumption from an individual smartphone usage [14]. However, the complexity of modern smartphone makes it difficult to accurately measure its energy consumption [5].

Here, a simple model was established to calculate the average daily power consumption, *E*_ad_, of a smartphone, under the two assumptions: (1) undergraduates charge their smartphones only when the battery nearly runs out; (2) heat loss of the battery is ignored.

$$E_{ad}=V\times C\times F$$ (1)

Where, *V* is the voltage of smartphone battery (i.e., 3.7 volt). *C* and *F* are the average battery capacity and charging frequency, respectively. Here, 1750 mAh (average of 1500 and 1999 mAh) and 1 time per day were used as the inputs of *C* and *F*, respectively, according to the survey results.

The average daily power consumption (i.e., *E*_ad_) from one smartphone is estimated to be 6.475 Wh. There are 37 million of on-campus undergraduates in China, in 2015. Thus, 239,575 kWh of electric energy will be consumed per day, due to their smartphone usage. As far as entire mobile phone system is concerned, energy consumption will be much more than that [14]. So, college student’s rational usage of their smartphone is an issue for green and sustainable university [5].

## Conclusions

This paper provides some understanding on sustainable development in higher education in western China, by investigating on-campus undergraduates’ smartphone usage and their pro-environmental behaviors in Xi’an, China. Most of the participated undergraduates had a fashion smartphone with a large screen and high battery capacity, which also had long daily standby/usage time and frequent charging. As far as the above cases are concerned, female students had higher percentages than their male peers. Average daily power consumption from a smartphone was estimated to be 6.475 Wh, according to the established model. On-campus undergraduates changed their smartphones frequently, which produced large quantities of WEEE annually.

Most respondents treated their waste smartphones in the proper ways, such as take-back service and trading with the peddler/second-hand market. However, some surveyed undergraduates were short of the basic environmental knowledge about smartphones. Some of them participated much less in on-campus practices and internships. Undergraduates should be instructed to use their smartphones rationally and prolong its lifespan. They also need to improve their knowledge, skills and environmental concerns about WEEE treatment. They should also be encouraged to participate in various environmental practices and environmental education as much as possible, in order to benefit their academic performance, mental and physical health, green consumption and sustainable lifestyles.

The findings of this work have various practical implications for on-campus undergraduates’ pro-environmental behavior in western China. It will also contribute to the sustainable development in Chinese higher education. However, this survey only covered part of universities in Xi’an, due to the limited sampling. From the viewpoint of environmental concerns, EEE usage of Chinese undergraduates will be comprehensively investigated in our future work, especially the effects of smartphone usage of on-campus undergraduates on their academic performance.

## Supporting information

S1 File Questionnaire response data. (XLS)

S1 Graphical abstract (TIF)
