# Supplementary figures and images for: Pro-environmental behavior and smartphone uses of on-campus engineering students in Xi’an, China
Source: PLoS One. 2021 Nov 4;16(11):e0259542. doi: 10.1371/journal.pone.0259542 (PMC8568102; doi:10.1371/journal.pone.0259542)

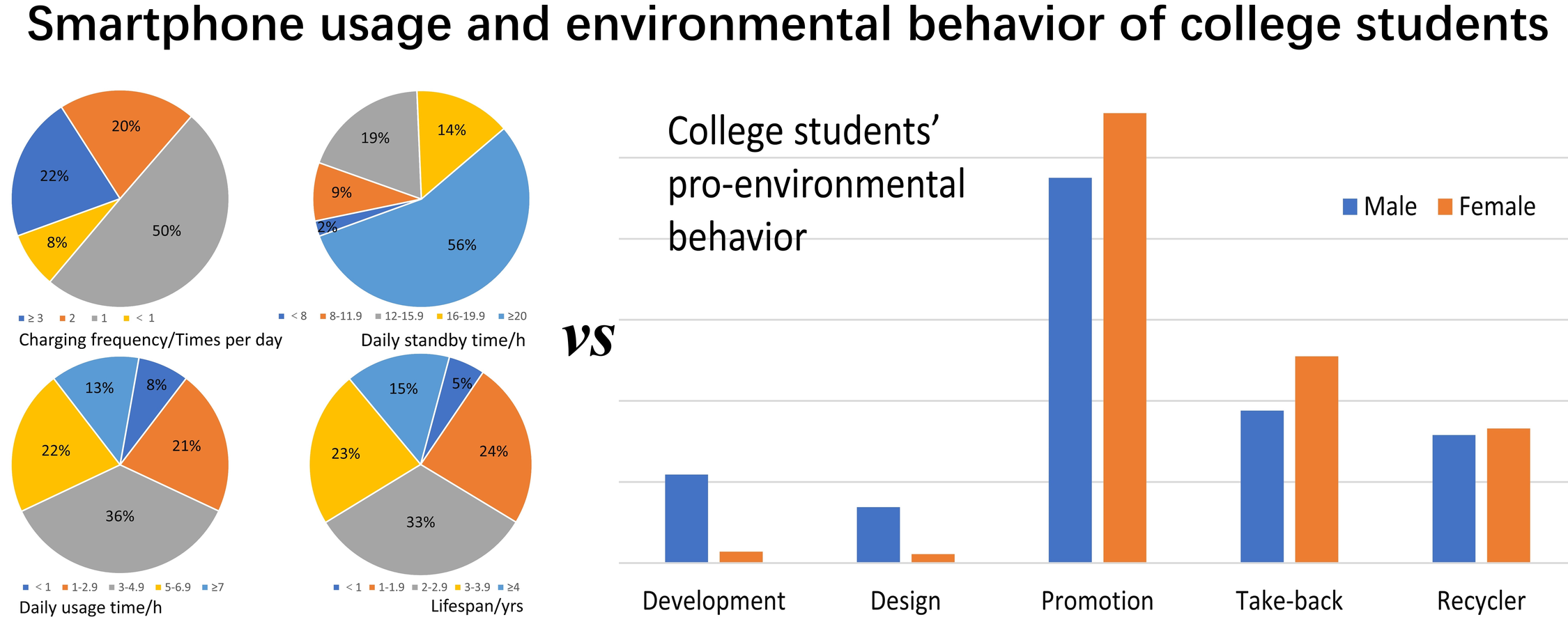

Supplement: S1 Graphical abstract — (TIF) [file pone.0259542.s002.tif]
